# The complete chloroplast genome of a Chinese endemic ornamental plant *Sorbus unguiculata* Koehne (Rosaceae)

**DOI:** 10.1080/23802359.2019.1624632

**Published:** 2019-07-11

**Authors:** Zhengyang Niu, Zhongren Xiong, Lianlian Xi, Xin Chen

**Affiliations:** Co-Innovation Center for Sustainable Forestry in Southern China, College of Biology and the Environment, Nanjing Forestry University, Nanjing, People’s Republic of China

**Keywords:** *Sorbus unguiculata*, ornamental, chloroplast genome, phylogenetic relationship

## Abstract

*Sorbus unguiculata* is a pinnate-leaved deciduous shrub growing in mixed forests in Sichuan province, China. It is one of the least known species in the genus with high ornamental value. In this study, we firstly assembled and annotated the complete chloroplast genome of the species using the next-generation sequencing method. The results showed that the chloroplast genome size is 159,900 bp, comprising of a large single-copy (LSC) region of 87,888 bp, a small single-copy (SSC) region of 19,256 bp, and a pair of IR regions of 26,378 bp. The whole cp genome predicted 109 genes in total, including 76 protein-coding genes, 29 tRNAs, and 4 tRNAs. The overall GC content of the chloroplast genome is 36.6%. The phylogenetic relationship exhibited that *S. unguiculata* is closely clustered with *S. helenae* in *Sorbus*.

*Sorbus unguiculata* Koehne is a beautiful shrub with small delicate leaves, white flowers, and fruits which are distributed in alpine regions of Sichuan province, China. It is one of the least known species in this genus, as no more information could be found except the initial morphological descriptions in the protologue. In this study, we firstly sequenced and assembled the complete chloroplast genome of *S. unguiculata* based on Illumina pair-end sequencing to provide more valuable genetic resources of pinnate-leaved taxa within *Sorbus* L.

Total genomic DNA was isolated from mature leaves of *S. unguiculata* using a modified CTAB method (Li et al. 2013). The samples were collected from conifer and broad-leaved mixed forests at the altitude of 3303 m in Balang mountain (101°58′31.51″E, 30°53′11.96″N), Sichuan, China. The voucher specimen was deposited at the Herbarium of Nanjing Forestry University (NF). The insert size of 270 bp fragments were enriched for the library construction, and the qualified reads was sequenced using the Illumina HiSeq 4000 platform. At last, 2G of high qualities raw data which have filtered adapters and low quality reads were generated. In the process of assembly, the clean reads were processed by GetOrganelle pipeline (Jin et al. 2018). The complete chloroplast genome was screened using the Bandage software (Wick et al. 2015). In order to obtain the accurate annotation, the chloroplast genome was transferred to Plastid Genome Annotator (PGA) software (Camacho et al. 2009) with the reference of *Sorbus torminalis* (NC_033975). The annotated chloroplast genome was artificially corrected in Geneious 9.1.4 (Kearse et al. 2012). Finally, the sequence was deposited into GenBank (accession number MK814479).

The complete chloroplast genome size of *S. unguiculata* was 159,900 bp, containing a large single-copy (LSC) region of 87,888 bp, a small single-copy (SSC) region of 19,256 bp, and two inverted repeat (IRa and IRb) regions of 26,378 bp. A total of 108 genes were detected in the chloroplast genome, including 76 protein-coding genes, 28 tRNA genes, and 4 rRNA genes. Interestingly, *rps12* was detected as a trans-spliced gene located in LSC and IR regions. The overall GC content was 36.6%, and GC contents in the LSC, SSC, and IR regions were 34.3%, 30.3%, and 42.7%, respectively.

The phylogenetic tree was established based on 76 protein-coding genes of 24 species with the software MAFFT v.7 (Katoh and Standley 2013), including 23 Maloideae species and one Rosoideae species (*Rosa roxburghii*) as the outgroup. The maximum likelihood (ML) analysis constructed by RAxML v8.0.0 (Stamatakis 2014) and Bayesian inference (BI) analysis set with MrBayes v3.2.2 (Ronquist et al. 2012) were combined to explore the systematic position of *S. unguiculata*. We discovered that *S. unguiculata* was clustered with *S. helenae* and formed the sister clade with *S. rufopilosa*, all of which belonged to the pinnate-leaved group (Figure 1). Besides, *S. tominalis* was embedded in *Malus* and *Chaenomeles*, which was not consistent with the ITS results (Xiang et al. 2017).

**Figure 1.**
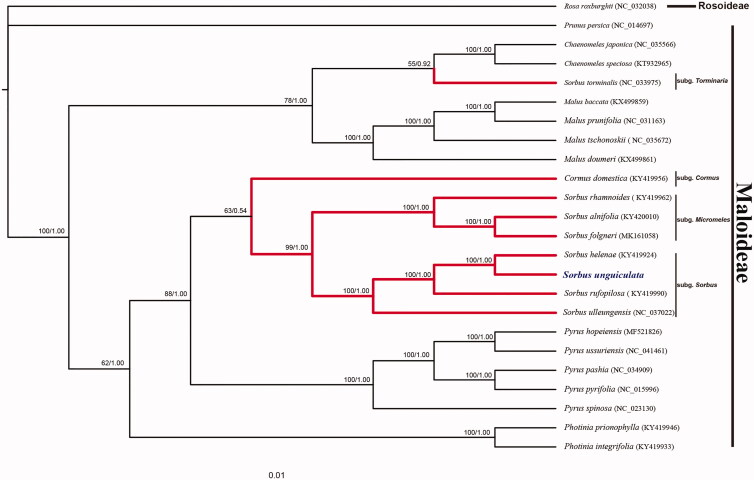
Maximum likelihood (ML) and bayesian inference (BI) method jointly introduced the phylogenetic relationship of *S. unguiculata* with 24 species based on 76 protein-coding genes. Numbers on the nodes were bootstrap values from 1000 replicates and the posterior probabilities values after 6,000,000 generations. *Rosa roxburghii* was selected as the outgroup.
